# Performance of Triple-Cation Perovskite Solar Cells under Different Indoor Operating Conditions

**DOI:** 10.1021/acsami.4c14736

**Published:** 2024-11-05

**Authors:** Marko Jošt, Žan Ajdič, Marko Topič

**Affiliations:** Faculty of Electrical Engineering, University of Ljubljana, Tržaška 25, 1000 Ljubljana, Slovenia

**Keywords:** perovskite solar cells, indoor operation, detailed balance limit, bandgap tuning, stability

## Abstract

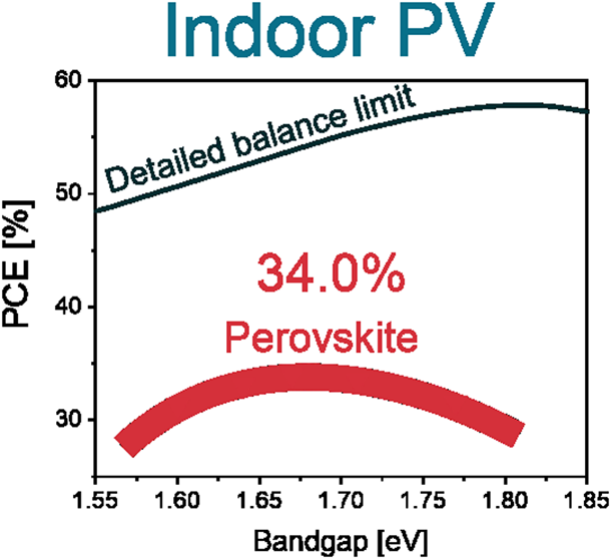

We systematically analyze triple-cation perovskite solar cells for indoor applications. A large number of devices with different bandgaps from 1.6 to 1.77 eV were fabricated, and their performance under 1-sun AM1.5 and indoor white light emitting diode (LED) light was compared. We find that the trends agree well with the detailed balance limit; however, the devices near the optimal bandgap (1.77 eV) perform worse due to the lower perovskite quality. Instead, we achieve the highest power conversion efficiency (PCE) of 34.0% under 870 lx with 1.67 eV and Al_2_O_3_ passivation. The perovskite with a bandgap of 1.71 eV is not far behind, with a high *V*_OC_ of 1.02 V. Measurements under different white LED color temperatures confirm that the highest PCE is achieved under the warmest colors. All measurements were carried out in a dedicated indoor setup that ensures the diffuse light typical of indoor environments and allows both short- and long-term measurements. In the best case, we observe no degradation during the 33-day test under simulated office conditions with regular switching on and off of the light and a *T*_80_ of 30 days under continuous illumination. The results were obtained from multiple batches, which corroborates our findings and gives them statistical relevance.

## Introduction

With the development of technology, our world is becoming more and more digitalized. Small electronic devices have found their way into all areas of our lives, and their number will continue to increase. The growth of Internet of Things (IoT) devices and applications also places high demands on their power supply. It is unrealistic for every device to have its own wiring to the electricity or its own battery. While the former causes high costs and high material consumption, the latter must be replaced or recharged at regular intervals. One of the most efficient solutions in this case is to use ambient light for their continuous power supply by exploiting photovoltaic devices.^1,2^

Perovskite solar cells are prime candidates for powering indoor electronics and IoT gadgets.^3,4^ They are highly efficient, while their tunable bandgap allows optimization according to the indoor illumination conditions. Importantly, indoor operation conditions are vastly different from the outdoor ones. Devices in field operation are regularly exposed to high temperatures and irradiance and also to diurnal and seasonal changes, in addition to high humidity and rain periods. All those factors massively affect perovskite stability, with the perovskite absorber being highly sensitive to all of them.^5^ Thus, indoor operation under low-light, constant mild temperatures (∼25 °C) without the possibility of rain presents ideal conditions for perovskite solar cells operation. Importantly, no potential hail damage to the module packaging and rain reduces the risk of lead egress into the environment.

The development of perovskite solar cells for indoor applications is similarly faster than for outdoor operation.^6−10^ However, the results are not always easily comparable due to different measurement conditions, such as illuminance (from 200 to 1000 lx) and spectra (white light emitting diode (LED) with different color temperatures or fluorescent).^11^ The current best device had a certified PCE of 44.7% under 1000 lx (338.2 μW cm^–2^) of fluorescent U30 lamp,^12^ owing to dual passivation by oleylammonium iodide solution in trichloromethane. Another highly efficient device was developed using double passivation with guanidinium- and phenylethylamine-based additives, resulting in a PCE of 40.1% under warm white LED with 824.5 lx and 301.6 μW cm^–2^.^13^ The devices reported by Li et al. achieved 42.1 and 39.2% illuminated by 3000 K white LED under 1000 (280 μW cm^–2^) and 500 lx, respectively.^14^ All reports show the high promise of perovskite solar cells for indoor operation and indicate that adjusted perovskite design is needed for optimal performance, as well as various measurement conditions applied.

In our recent paper, we have analyzed the long-term performance of perovskite solar cells under real indoor conditions.^15^ The devices were not optimized for indoor operation under LED lighting, utilizing a lower 1.63 eV bandgap. The best device, encapsulated with edge-sealant, has survived almost a year without meaningful degradation. However, light soaking effect has been observed, similar to the one observed outdoors.^16^

In this paper, we investigate the requirements for optimal indoor operation and analyze the performance of perovskite solar cells with different bandgaps. First, we analyze the detailed balance limit (DBL) using typical indoor lighting spectra in search of the optimal perovskite bandgap in terms of maximum energy yield. We test this by fabricating a large number of perovskite solar cells with different bandgaps ranging from 1.6 to 1.77 eV with and without Al_2_O_3_ passivation. We systematically analyze their performance and PV parameters under different indoor illumination conditions as well as under AM1.5. For the short- and long-term measurements, a dedicated indoor setup is developed and described to be used in experiments.

## Results

**Figure 1:**
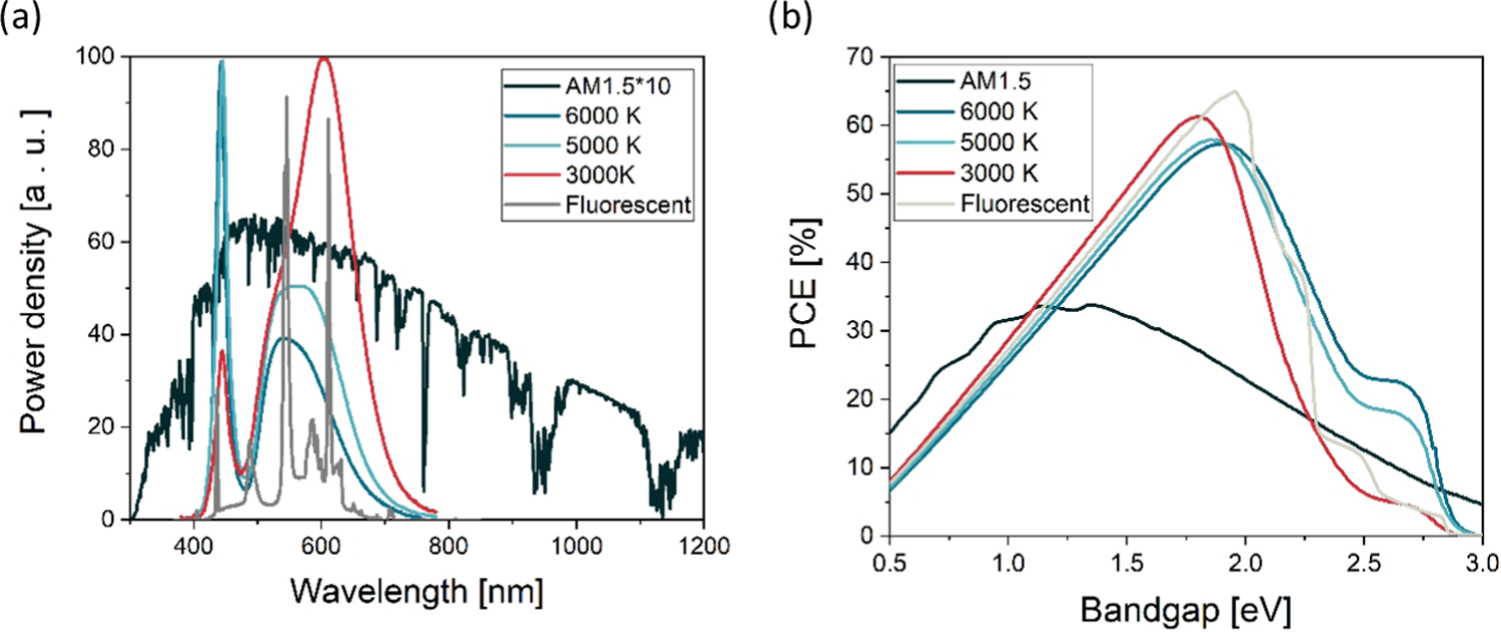
(a) Comparison of AM1.5 spectrum and illumination spectra of different indoor light sources. (b) Detailed balance limit calculations for different spectra.

Figure 1a shows a comparison between the standard spectrum of the sun outdoors, AM1.5, and typical indoor spectra of white LEDs with different color temperatures and a fluorescent bulb. A clear difference can be seen, particularly in the near-infrared range, whereby most spectra of indoor light sources only emit light up to 800 nm. This means a clear advantage for the use of perovskite solar cells, with typical absorption onsets around 800 nm (1.6 eV), resulting in lower thermalization losses and almost no near-infrared losses. Consequently, higher PCEs as well as output power are expected. Figure 1b shows the calculation of the detailed balance limit (DBL)^17^ for the spectra in Figure 1a. The peaks for the AM1.5 spectra are at 1.1 and 1.4 eV with a theoretical PCE maximum of 33.5%. However, due to a different spectrum, the maximum achievable PCE under indoor illumination exceeds 60%, with the optimal bandgap range being between 1.75 and 2 eV depending on the light source. A higher spectral peak at 600 nm for the 3000 K white LED results in a lower optimal bandgap at 1.8 eV compared to colder white LEDs (5000+ K), which have a peak at 1.86 eV and above. The former also have a higher PCE potential due to lower thermalization losses. DBL analysis shows that devices with, for example, bandgap below 1.6 eV are not optimal for indoor operation under common LED light sources. Therefore, conventional silicon solar cells and their tandems are not suitable for indoor applications. Instead hydrogenated amorphous silicon, which is already an established solution,^2,18^ perovskite or perovskite/perovskite tandem solar cells can be used.

### Indoor Measurement Setup

**Figure 2:**
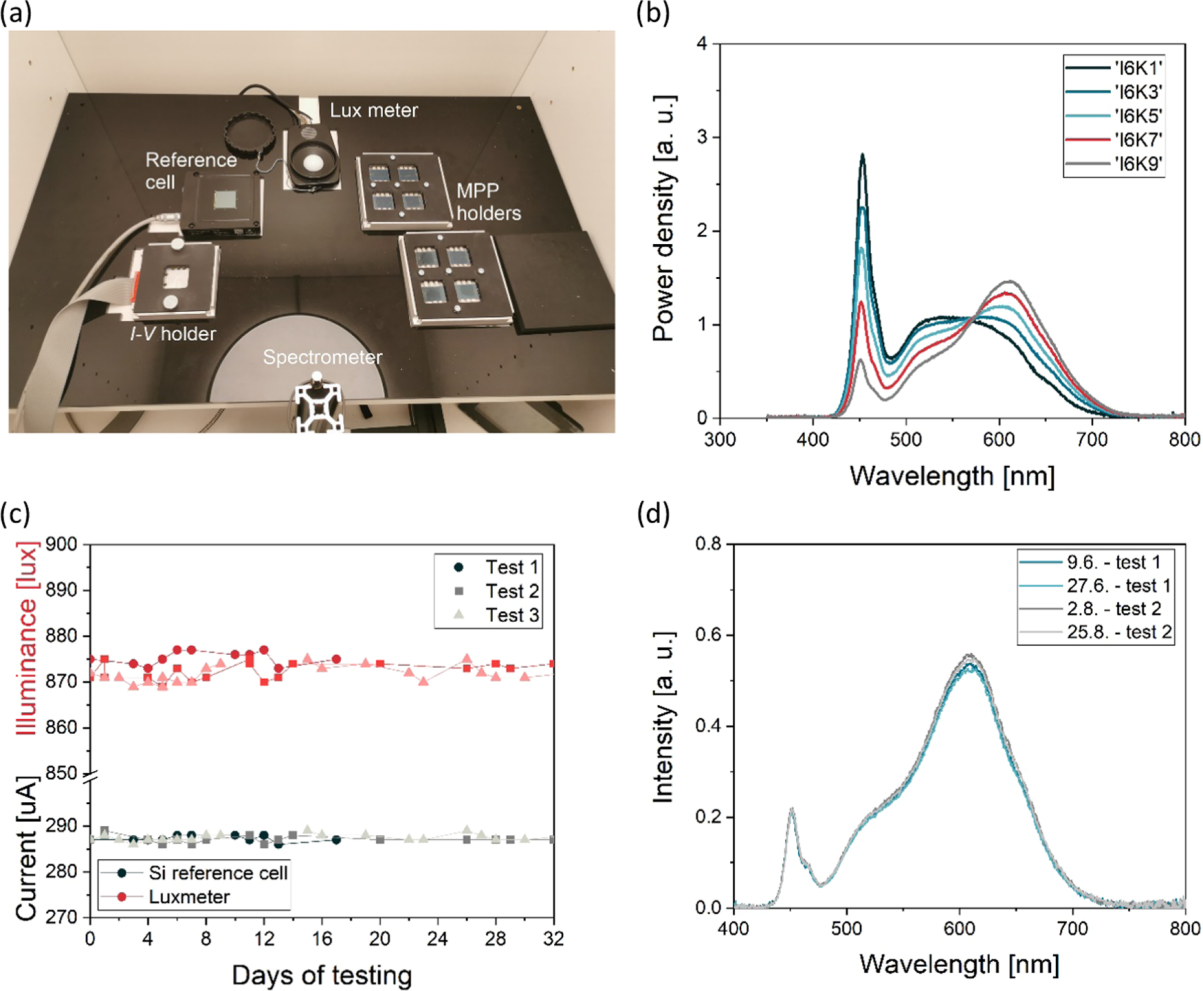
(a) Photograph of the measurement setup with an LED lamp, white walls, and black bottom. The measurement equipment is denoted. (b) different spectra (‘K1, 3, 5, 7, and 9′’) obtainable with the LED lamp for the sixth intensity (‘I6′). (c) Stability of the lamp measured with reference solar cell and luxmeter during three different tests in a period of 17 and 32 days, respectively. (d) Stability of LED lamp spectrum, measured twice during two tests.

For testing perovskite solar cells under indoor conditions, we have developed a setup (based on the recommendations by Michaels et al.^19^) with diffuse illumination. The setup is in a white closet with a commercial white LED ceiling lamp as a light source and the black bottom platform for devices under test (Figure 2a). The lamp can tune the intensity and color of white LED in up to 10 levels. The possible spectra of the white LED are shown in Figure 2b. To test the stability of the lamp and determine the correct intensity during the measurement, the setup is equipped with a KG5 silicon reference solar cell, a luxmeter, and a spectrometer. These instruments and the devices under test are arranged in a circle around the center of the lamp to ensure the intensity is the same at every spot (±1%). To reduce the effects of reflection on the readings of the sensors, the measurement plate is covered with a black Plexiglas, as shown in the photo in Figure 2a. The measurements over a test period of more than 3 weeks have shown great stability of the light source (Figure 2c,d). Both the reference solar cell and the luxmeter showed a change of less than 1% during the test period, and the spectrum also only marginally changed within the uncertainty range. This shows that despite the lack of feedback control of the lamp, the test conditions in the designed setup are stable and reliable. The temperature during the test was in the range of 24–27 °C without cooling or ventilation in the closet since the intensity of the lamp is so low and its efficiency so high that it does not cause any heating. Such a small temperature deviation does not impact the solar cell due to the low temperature coefficient of perovskite solar cells.^20^ There are three sample holders in the setup. One (bottom left) is intended for *I–V* measurements, while the two on the right can accommodate up to 4 samples each and are intended for long-term stability measurements. All samples are connected to the MPP tracking system. Further photos of the system and the analysis of the lamp are shown in Figures S1, S2, and S3. Excluding the commercial light source, for which we presented detailed characterization of spectra, intensity, and stability, our setup follows the recommendations of the IEC TS 62607–7–2:2023 standard for indoor measurements of PV devices. As a result, we obtain an excellent match between the *J*_SC_ from *I–V* and EQE measurements as will be shown later.

### Solar Cell Results

Based on the DBL analysis, which recommends perovskite solar cells with wider bandgap, we have focused on bromide-rich devices. As a base, we chose a typical triple-cation perovskite, in which CsI is added to the so-called MAFA perovskite.^21,22^ Different bandgaps were obtained by mixing MaPbBr_3_ and FAPbI_3_ in different ratios. As extracted from the infliction point of EQE measurements,^23^ this resulted in bandgaps of 1.6, 1.64, 1.67, 1.71, and 1.77 eV. The fabricated perovskite solar cells have a full layer stack: glass|ITO|MeO-2PACz|perovskite|C_60_|SnO_2_|Cu, with an active area of 0.17 cm^2^. To improve the short-term performance, a thin, 1 nm thick Al_2_O_3_ was deposited on the perovskite absorber. Similarly, to improve long-term performance, all devices were capped with a 30 nm Al_2_O_3_ layer, both of which we described in more detail in our previous report.^24^

**Figure 3:**
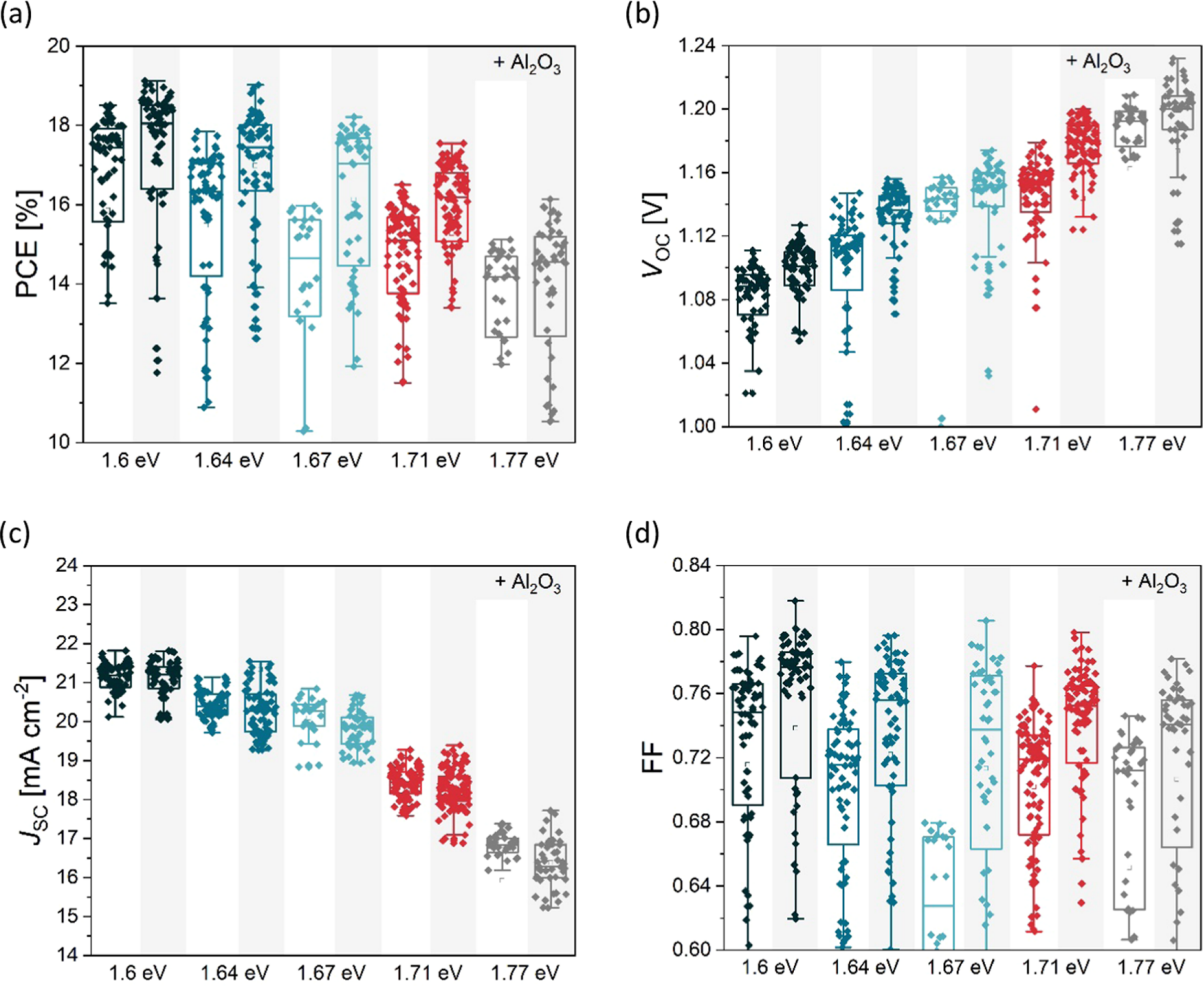
PV performance parameters of fabricated perovskite solar cells with different bandgaps under simulated 1-sun conditions. (a) PCE, (b) *V*_OC_, (c) *J*_SC_, and (d) FF. Cells with a 1 nm Al_2_O_3_ interlayer for each composition are also denoted in the graph.

**Figure 4:**
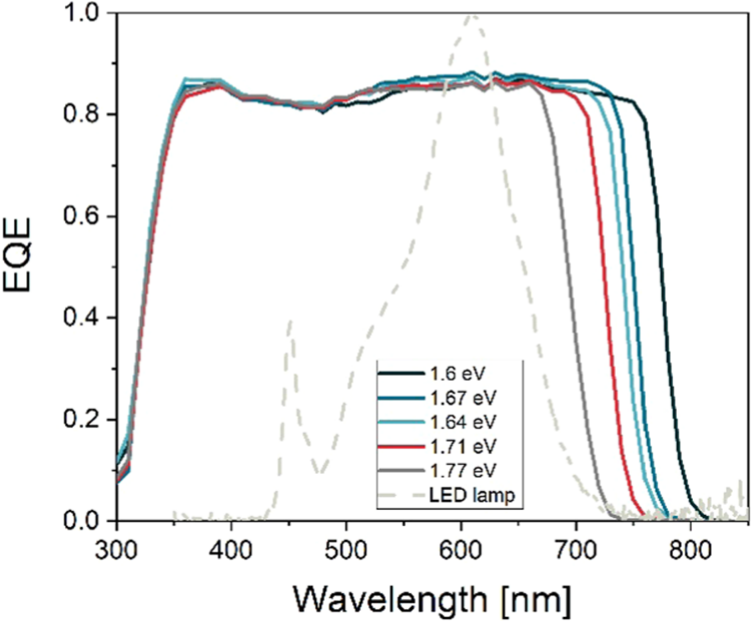
EQE measurements of fabricated devices with different bandgaps. The integrated *J*_SC_ values for 1-sun AM1.5 and indoor illumination are shown in Table 1. The LED lamp spectra are also shown.

Figure 3 shows the PV performance parameters of more than 60 substrates (6 devices per substrate) from 8 batches, measured under simulated 1-sun, AM1.5 illumination (100 mW cm^–2^). The performance trends with respect to the different bandgaps are consistent with the DBL (Figure S4). Devices with higher bandgap have lower short-circuit current density (*J*_SC_) and higher open-circuit voltage (*V*_OC_), while PCE also decreases with increasing bandgap. The *J*_SC_ trends are confirmed by the EQE measurements (Figure 4 and Table 1). The absorption onset follows the change in bandgap and the integrated *J*_SC_ agrees very well with the *J*_SC_ from the *I–V* measurements, showing less than 1% difference. The *V*_OC_ reaches high values above 1.2 V for devices with 1.71 and 1.77 eV bandgaps. Such a high *V*_OC_ is enabled by the use of a 1 nm thick Al_2_O_3_ passivation layer, boosting both *V*_OC_ and especially the fill factor (FF) for all devices. Importantly, Al_2_O_3_ helps to improve performance to a standard expected level, even if the reference cells in the same batch were poor. This is especially the case at 1.67 eV, where the mean PCE is improved by more than 2% absolute with the use of Al_2_O_3_, mainly due to the improvement in FF: reference cells have an FF of below 70%, while cells with Al_2_O_3_ have a high FF of 75% and above. Overall, the Al_2_O_3_ improves the PCE by more than 1% absolute, resulting in PCEs of over 19% for the best devices with bandgaps of 1.6 and 1.64 eV.

**Table 1:** *J*_SC_ Calculated from EQE Measurements for Perovskite with Different Bandgaps^a^

	1.6 eV	1.64 eV	1.67 eV	1.71 eV	1.77 eV
*J*_SC_AM1.5_ [mA cm^–2^]	21.6	20.6	20.0	18.8	17.1
*J*_SC_LED_ [μA cm^–2^]	109.8	111.5	110.8	109.4	106.2

a First row shows values with AM1.5 spectrum, while the second is for the indoor lamp used in this paper.

**Figure 5:**
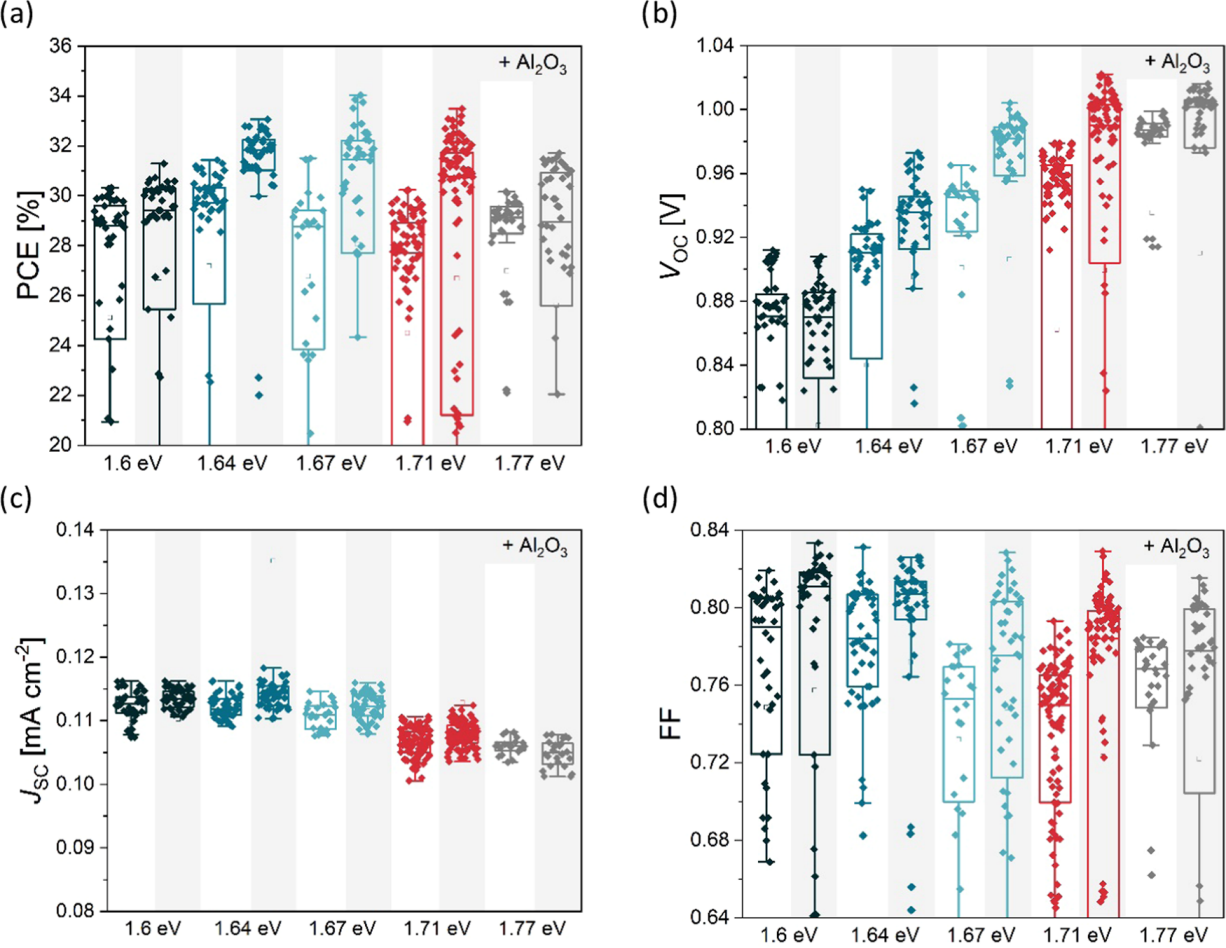
Indoor PV performance parameters of solar cell with different bandgaps under white LED at 870 lx. (a) PCE, (b) *V*_OC_, (c) *J*_SC_, and (d) FF. Cells with 1 nm Al_2_O_3_ interlayer for each composition are also denoted in the graph with the shaded area behind it.

Same devices were afterward tested under indoor conditions in the above-described indoor setup. We have selected the second lamp intensity with 870 lx (287 μA from the reference cell) and the warmest color, which corresponds to irradiance of 272 μW cm^–2^. The results again show increased *V*_OC_ in the devices with higher bandgap; however, due to the LED spectra limited to below 700 nm, we do not see such changes in *J*_SC_ as in the case of AM1.5 spectra. Thus, the *J*_SC_ is quite constant for all of the combinations (±1%), except for the slightly lower *J*_SC_ of the 1.77 eV device, where its absorption onset already crosses the LED spectrum. The *J*_SC_ from *I–V* and EQE measurements match well again (Figure 5 and Table 1), confirming that determination of LED lamp spectra and power was correct.^11^ The devices with 1.71 eV reach exceptionally high *V*_OC_ values above 1.02 V, owing to Al_2_O_3_ passivation. Interestingly, the devices with a higher 1.77 eV bandgap have lower *V*_OC_ under indoor conditions despite comparable *V*_OC_ under 1-sun AM1.5. This is most likely a result of lower solar cell quality and performance, as visible in Figure 3 and especially FF graphs. The Al_2_O_3_ passivation again significantly improves device performance, in some cases, the *V*_OC_ is improved by 40 mV. We thus show that Al_2_O_3_ passivation is a suitable solution for outdoor and even more for indoor conditions. Overall, the best PCE of 34.0% was obtained with a 1.67 eV device, but the best device with a bandgap of 1.71 eV is not far behind, only trailing due to lower *J*_SC_.

In Figure S4, we compare the results obtained under 1-sun AM1.5 and indoor spectra with the DBL. In general, the results under the 1-sun AM1.5 spectrum follow the trends better. The bandgap to *V*_OC_ loss slightly increases with the bandgap and the FF decreases with the bandgap; however, PCE and the *J*_SC_ trends are in line with the DBL. For the indoor spectra, we observe larger discrepancies, especially for the device with a wide bandgap of 1.77 eV, where the measured *V*_OC_, and consequently also the PCE, deviate strongly from the expected value. Contrary to the 1-sun AM1.5 case, the FF stays with the Al_2_O_3_ passivation constant around 82%. We postulate the *V*_OC_ trends stem from using the same hole and electron selective contact layers for all devices that have larger misalignment with increasing perovskite bandgap.^25,26^

The best, 34.0% device was then measured under different spectra and intensities of the LED lamp (Figure S2). This allowed us to analyze their effects on the PCE. The lowest intensity was 250 lx, and the highest 3900 lx. The results shown in Figure S5 reveal some trends. We can see that as the light intensity increases, the PCE also increases. This is due to the logarithmic increase in *V*_OC_ with light intensity and, in the case of the transition from intensity 1 to 2, also due to the increase in FF. We hypothesize that the higher charge carrier density fills some of the defects and the lowest intensity is not sufficient in our case. Nevertheless, when we plot the *V*_OC_ versus the logarithmic *J*_SC_, we obtain a linear dependence and an ideality factor of 1.47 (Figure S6), which are expected for perovskite solar cells. Finally, warmer LED light leads to higher PCE, as predicted by the DBL. The PCE difference between the coldest and warmest light can be up to 5% absolute, yet the devices produce similar power, only the thermalization losses are lower in the case of the warmer white light.

Our record device is comparable to one of the best published results, the 40.1% device by He et al.^13^ The *V*_OC_ of 0.995 V is only slightly behind its 1.008 V, while our FF of 82.0% is higher than their 79.5%. The largest discrepancy is in *J*_SC_. Our device generates only 114 μA cm^–2^ at 870 lx (273 μW cm^–2^) almost 25% less than the 152 μA cm^–2^ (824 lx, 307 μW cm^–2^) generated by the device from He et al. The lower number of lux at higher incident power suggests that the lamp they used produces even warmer white light than ours; however, they did not provide any spectra. This emphasizes that warm white light yields the highest PCEs, the importance of reporting the spectra, and how small changes in spectra can cause large differences in indoor PCE values.

**Figure 6:**
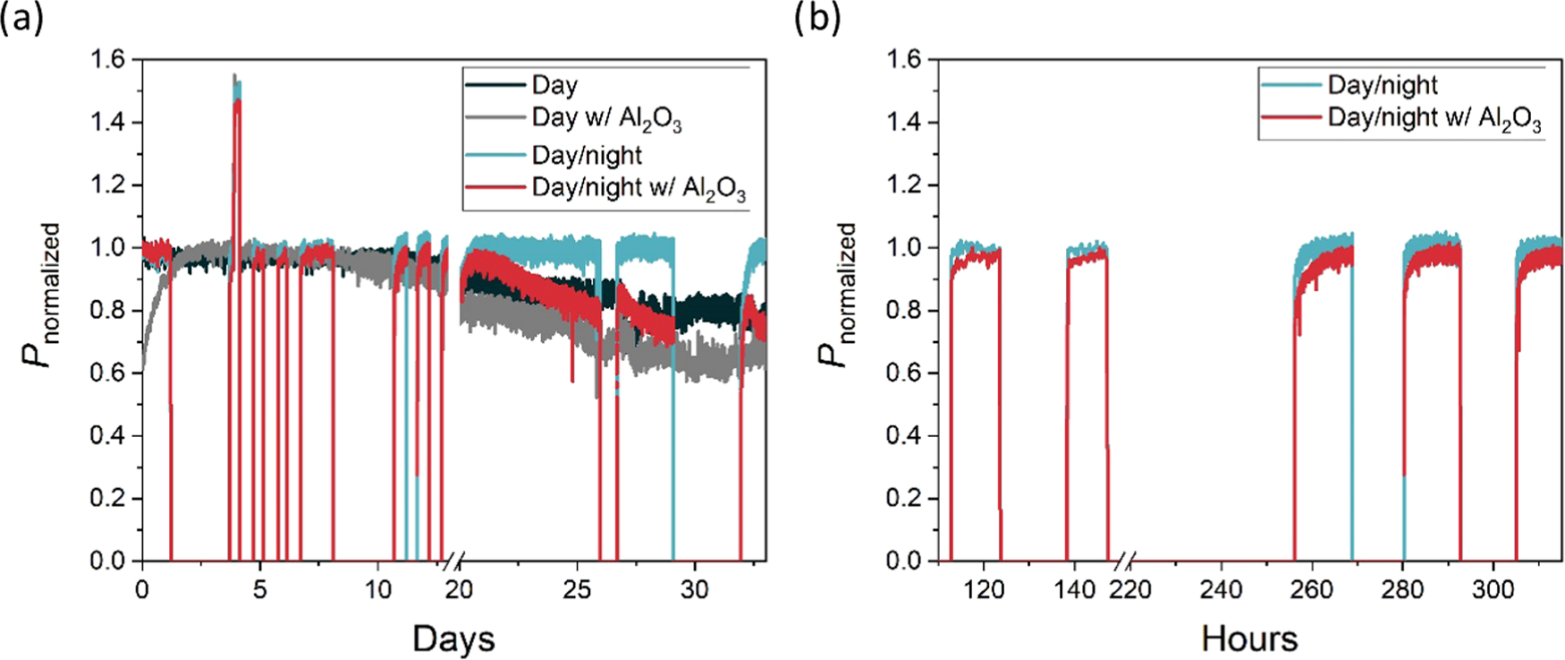
(a) MPP tracking data for cells with 1.71 eV during 33 days of tracking. Two cells with Al_2_O_3_ and 2 cells without it were tested under continuous and cyclic illumination. (b) Zoom-in of the MPP tracking focusing on a couple of days with a clear light soaking phenomenon.

Finally, we tested the stability of the devices. The devices were placed in the developed setup and connected to the MPP tracking system for long-term monitoring. We tested the devices under continuous and cyclic illumination. The cyclic illumination was performed by covering the samples in the evening before leaving the workplace and uncovering them in the morning. Thus, the illumination time varied from day to day, and no illumination was used at the weekends. In this way, the conditions of the office operation were simulated. The MPP tracking results for devices with 1.71 eV from a 33-day test are shown in Figure 6. Good stability can be observed. In the best case, the device without Al_2_O_3_ shows no degradation after 30 days of cyclic illumination. The devices with Al_2_O_3_ are slightly less stable, under continuous and cyclic illumination, in line with our previous results.^24^ Degradation is only observed after a long period of illumination (day 20 to day 25). After a short break of one night, the performance increases slightly and then decreases again. This meta-stability has been observed before^16,27^ and is attributed to ion migration, and influenced by cumulative irradiance and temperature. Interestingly, although Al_2_O_3_ initially passivates the perovskite/C_60_, it accelerates the effect of ion migration with time, resulting in poorer long-term stability and a longer light soaking time. In Figure 6b, we show a zoomed-in graph, focusing on a few days during the MPP tracking. At the beginning, between 115 and 150 h, light soaking is completed quickly. Later in the test, between hours 250 and 320, it takes much longer for the device to become completely soaked with light. In the worst case, it can take more than 12 h under indoor conditions (low light, room temperature), as shown by the red curve (Al_2_O_3_-passivated device) around 260 h. This day was an extreme case as the light was switched off 50 h before. The next day, light soaking is completed much faster. The results show the dynamic behavior of light soaking. The performance of the device is not only influenced by the phenomenon itself, but the speed also depends on the previous history of the device. Although it is to be expected that it takes longer to light soak an aged device, our results show that light soaking occurs faster after a period of illuminated days than after a period of dark days. Consequently, further in-depth analysis of the behavior of light soaking is required.

In Figure S7, we analyze the daily degradation and light soaking losses of the devices tested under cyclic illumination. The worst performing device is the device with a bandgap of 1.77 eV. Besides slightly lower PCE than expected, it also shows stronger degradation and is more prone to light soaking. Other bandgaps show little degradation over the test period of 33 days, as well as lower light soaking losses. Despite the light soaking phenomenon being visually easily observable in the MPP tracks during light cycling, the daily energy lost due to it is typically only around 5%.

## Conclusions

In this paper, we compared the performance of triple-cation perovskite solar cells with different bandgaps under standard AM1.5 and indoor LED illumination. Five different bandgaps of perovskite absorber with and without Al_2_O_3_ passivation layer were tested, and the results fit well with the detailed balance limit (DBL). Under AM1.5G, the absorber with the lowest bandgap of 1.6 eV performed best, achieving a PCE above 18 and 19% without and with Al_2_O_3_, respectively. As the bandgap increases, the *J*_SC_ decreases and *V*_OC_ increases, reaching more than 1.2 V for 1.7 and 1.77 eV perovskites. The performance trends under LED illumination are different. The best performance is shown by the device with 1.67 eV and Al_2_O_3_ passivation, which achieves 34% with *V*_OC_ > 1 V and FF above 82% under 870 lx (273 μW cm^–2^). While the DBL predicts optimal performance at higher bandgaps around 1.8 eV, the PCE of our device with a wide bandgap of 1.77 eV is lower, as we see a slight drop in *J*_SC_ and no expected gain in *V*_OC_ at higher bandgap. In total, more than 300 devices fabricated in multiple batches were analyzed, demonstrating the statistical relevance of our results.

The developed indoor measurement system allows accurate measurements, which were confirmed by the excellent agreement between the *J*_SC_ from *I–V* and EQE measurements as well as long-term stability measurements that utilize the excellent stability of the commercial LED ceiling lamp. The best cell showed no degradation after 33 days of testing under cyclic illumination, while the device under continuous illumination degraded by 20%. We also observed a strong light soaking effect, which increases with the duration of the test. The effect is more pronounced when the device has had a few days break beforehand. Therefore, the short-term history of the device is as important as the long-term history when it comes to analyzing light soaking.

## Experimental Section

### Materials

The FAI (formamidinium iodide) and MABr (methylammonium bromide) were purchased from Dyenamo, PbI_2_, PbBr_2_, and MeO-2PACz from TCI, and CsI from abcr GmbH. C_60_ was purchased from Creaphys and copper from Umicore. The ALD precursors were all bought from Strem. Anhydrous DMSO (dimethyl sulfoxide), DMF (dimethylformamide), and ethyl acetate were purchased from VWR. ITO-coated glass substrates with a sheet resistance of 15 Ω·sq^–1^ were purchased from Automatic Research.

### Device Fabrication

The layer stack of the fabricated perovskite solar cell is glass|ITO|MeO-2PACz|perovskite|C_60_|SnO_2_|Cu. In some devices, an Al_2_O_3_ passivating layer was deposited via ALD on top of perovskite. Each substrate accommodates 6 solar cells with an active area of 0.17 cm^2^.

The ITO substrates are cleaned in an ultrasonic bath in a four-step (acetone, 2% mucasol, DI water, and isopropanol) procedure, followed by 15 min in a UV ozone cleaner. MeO-2PACz is prepared by dissolving 0.3 mg of powder in 1 mL of ethanol. Perovskite preparation starts by dissolving PbI_2_ and PbBr_2_ in DMF/DMSO (4:1) solvent, while CsI is dissolved in pure DMSO (all 1.5 M solutions). Then, PbI_2_ solution is added to the FAI powder and PbBr_2_ solution to the MABr powder. Perovskite is then formed by mixing FAPbI3 and MAPbBr3 solutions in desired ratios (83:17, 77:23, 75:25, 70:30, and 60:40 in our case) and adding 5% vol. of CsI solution. Details were published in ref (22).^22^

MeO-2PACz (SAM2) is spin-coated at 3000 rpm for 30 s and annealed at 100 °C for 10 min. The perovskite layer is spin-coated at 4000 rpm for 35 s. After 25 s, the films are washed with 0.4 mL of ethyl acetate as antisolvent. The films are annealed at 100 °C for approximately 35 min. 21 nm of C_60_ is evaporated at a rate of around 0.1 Å/s. SnO_2_ and Al_2_O_3_ layers are deposited by atomic layer deposition (ALD) using the GEMSTAR tool from Arradiance. 20 nm of SnO_2_ layer is deposited at 80 °C using H_2_O and TDMASn (Tetrakis(Dimethylamino)Tin) precursors. Layers of Al_2_O_3_ (1 nm for passivation or 30 nm for capping) are deposited using precursors of H_2_O and TMA (trimethylaluminum) at 100 °C. The devices are exposed to air before the ALD deposition. Finally, 100 nm of copper as a back contact is evaporated at a rate of 1 Å/s through the mask to define the active area.

### Measurements

The current–voltage (*I–V*) curve was measured using a Keithley 2400 Source Meter Unit in air with a scan rate of 0.25 V s^–1^ and a step of 20 mV. One sun measurements were carried out under the illumination of simulated AM 1.5 G solar light from a Newport solar simulator system, class ABA. Indoor measurements were carried out under a commercial 24 W LED lamp with a diameter of 36 cm, bought in a local shop (catalog number ZM5165).

MPP tracking was performed in the custom-built measurement setup, described using a custom-built MPP tracking system.

EQE measurements were performed using a xenon light and Oriel monochromator system from Newport. EQE was measured as a function of wavelength from 300 to 850 nm with a step of 10 nm.

Fabrication steps were performed in a nitrogen glovebox (spin-coating) or in vacuum (evaporation, ALD). All measurements were performed in air.

## Supporting Information Available

Detailed characterization of the used LED lamp as well as measurements of champion device under different light intensities and spectra of the LED lamp (PDF)
