# Supplementary material for: Performance of Triple-Cation Perovskite Solar Cells under Different Indoor Operating Conditions
Source: ACS Appl Mater Interfaces. 2024 Nov 5;16(45):62195–202. doi: 10.1021/acsami.4c14736 (PMC11565567; doi:10.1021/acsami.4c14736)
Supplement: Supplementary file 1 — am4c14736_si_001.pdf [file am4c14736_si_001.pdf]

Supporting information

Performance of Triple-Cation Perovskite Solar Cells under Different Indoor Operating Conditions

Marko Jošt*, Žan Ajdič and Marko Topič
Faculty of Electrical Engineering, University of Ljubljana, Tržaška 25, 1000 Ljubljana, Slovenia
*Corresponding author: marko.jost@fe.uni-lj.si

LED lamp properties

a) b)

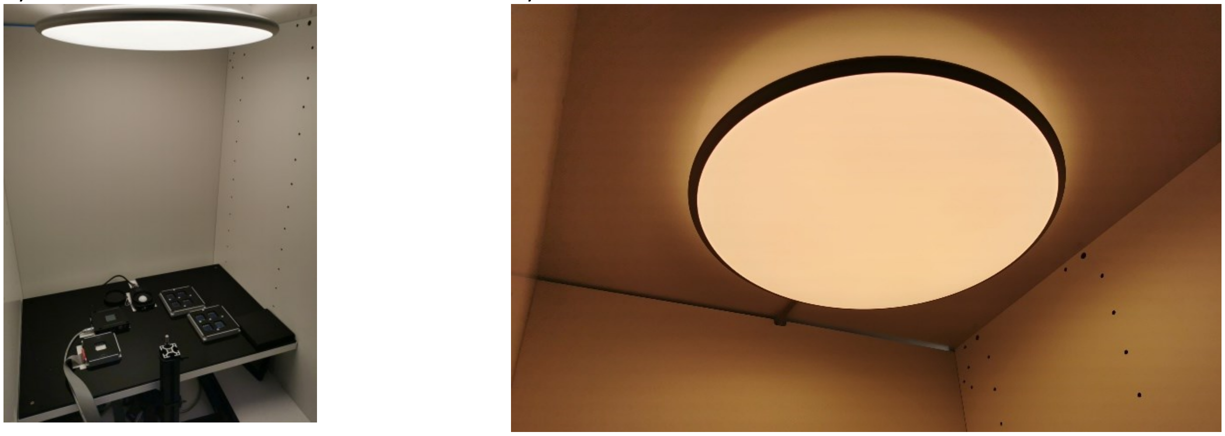

Figure S1: a) photograph of the setup including the lamp and all the instruments. B) a close up photograph of the used LED lamp

a) b)

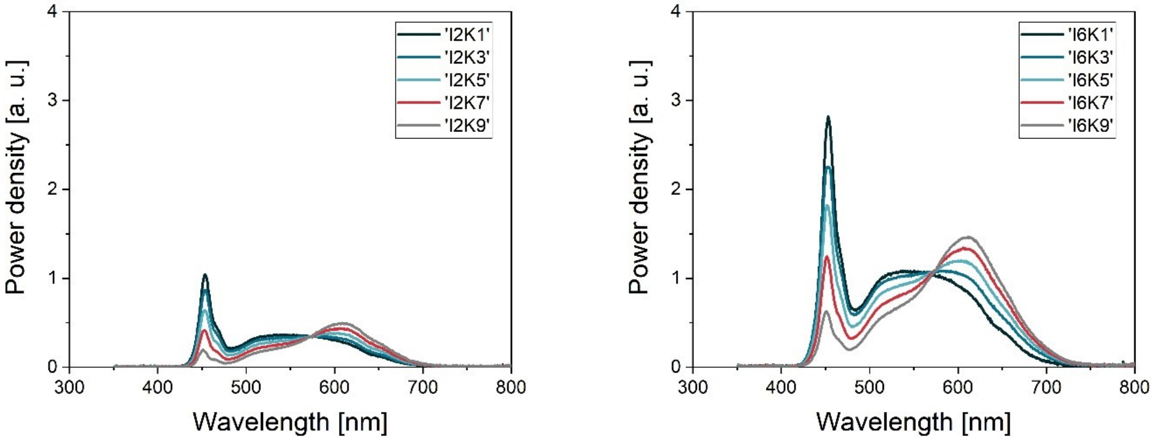

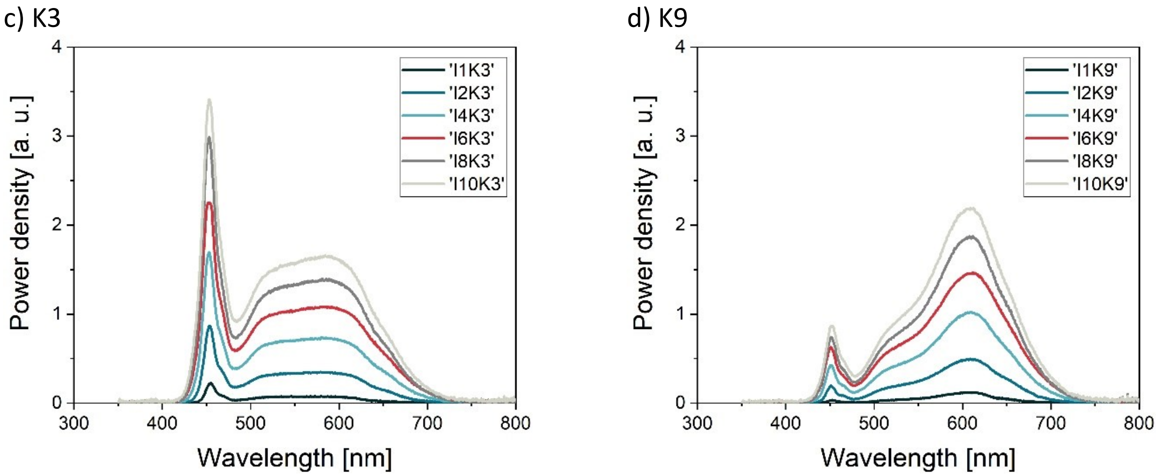

Figure S2: spectra of the LED lamp measured at different intensities and colors of light. The former is indicated in the legend with an "I", while the latter with "K". I2K7 means second intensity, seventh color (warm) setting of the lamp. a) is second intensity, b) sixth intensity, c) third color and d) ninth color.

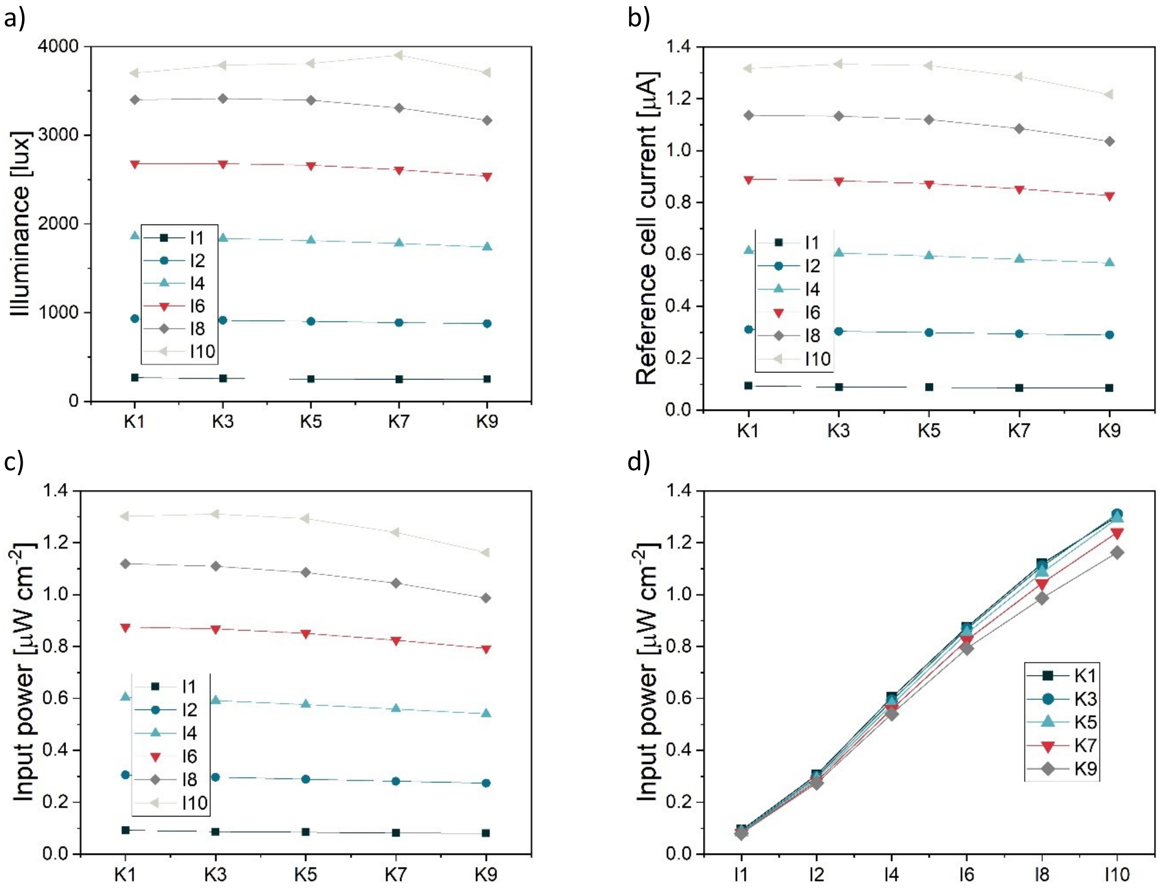

Figure S3: a) Illuminance in lux measured with a luxmeter at different intensities and colors of the LED. b) current from the reference KG5 solar cell at different intensities and colors of the LED. c) and d) input power at different intensities and colors of the LED. The intensity level is indicated by an "I", while the color of light with "K". I2K7 means second intensity, seventh color (warm) setting of the lamp.

Fabricated solar cells results versus DBL

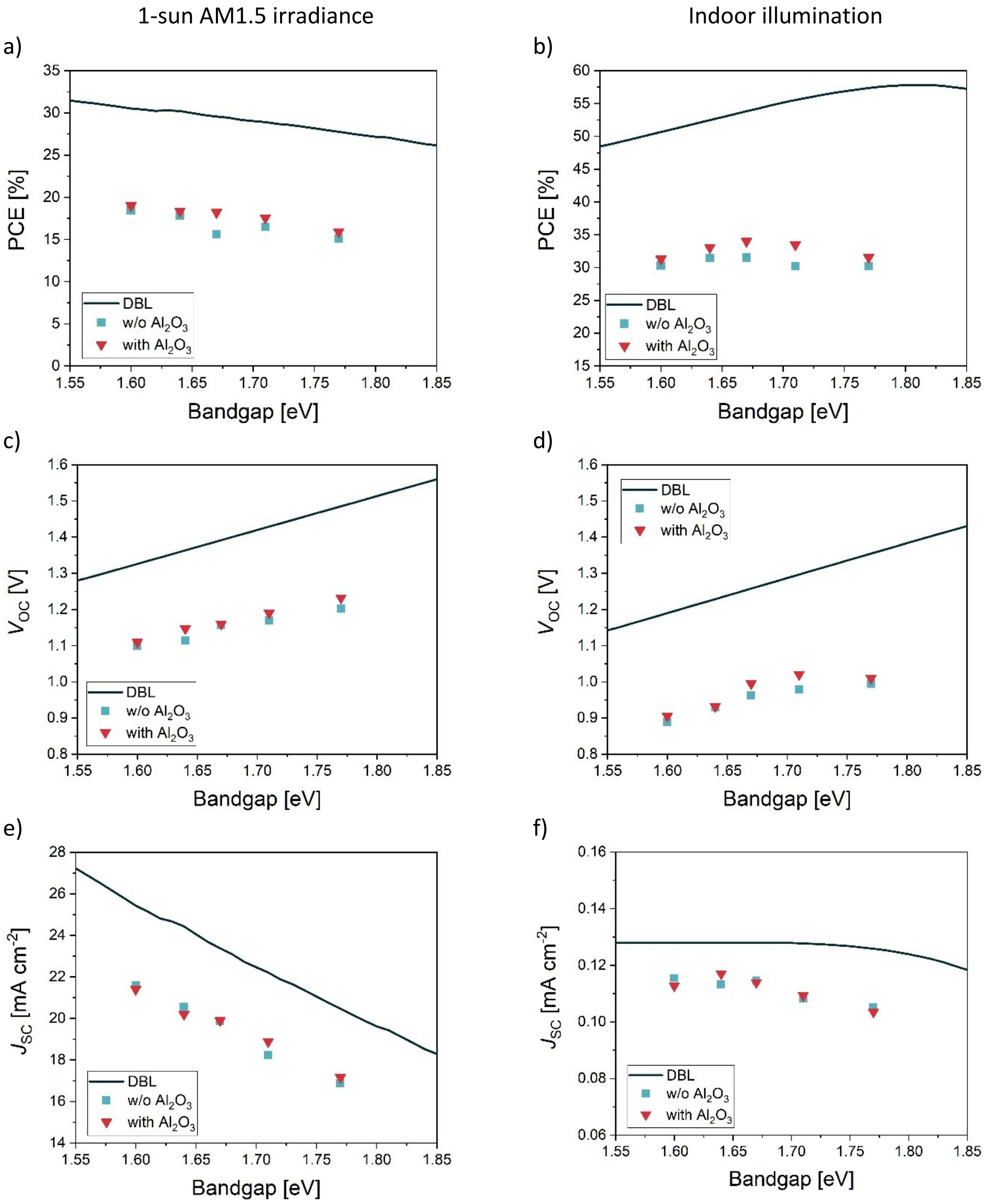

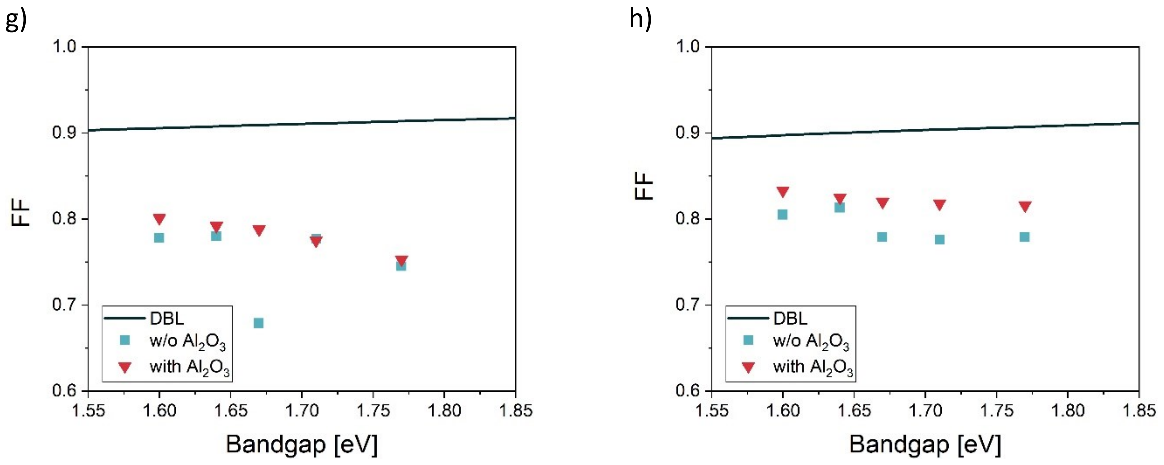

Figure S4: Comparison between main PV parameters (a) PCE, b) V_{OC}, c) J_{SC} and d) FF) of the best fabricated perovskite solar cell with their detailed balance limit (DBL) values for each bandgap. The left side graphs show comparison with the AM1.5 spectra, while the right side with the indoor spectra of our lamp. J_{SC} follows the DBL best, while biggest discrepancy is visible for FF, which should increase with bandgap, however, it decreases. The V_{OC} shows the predicted increase with the bandgap, however, not as steep. Thus the V_{OC} deficit increases with the bandgap.

Performance under different light intensities and spectra of the LED lamp.

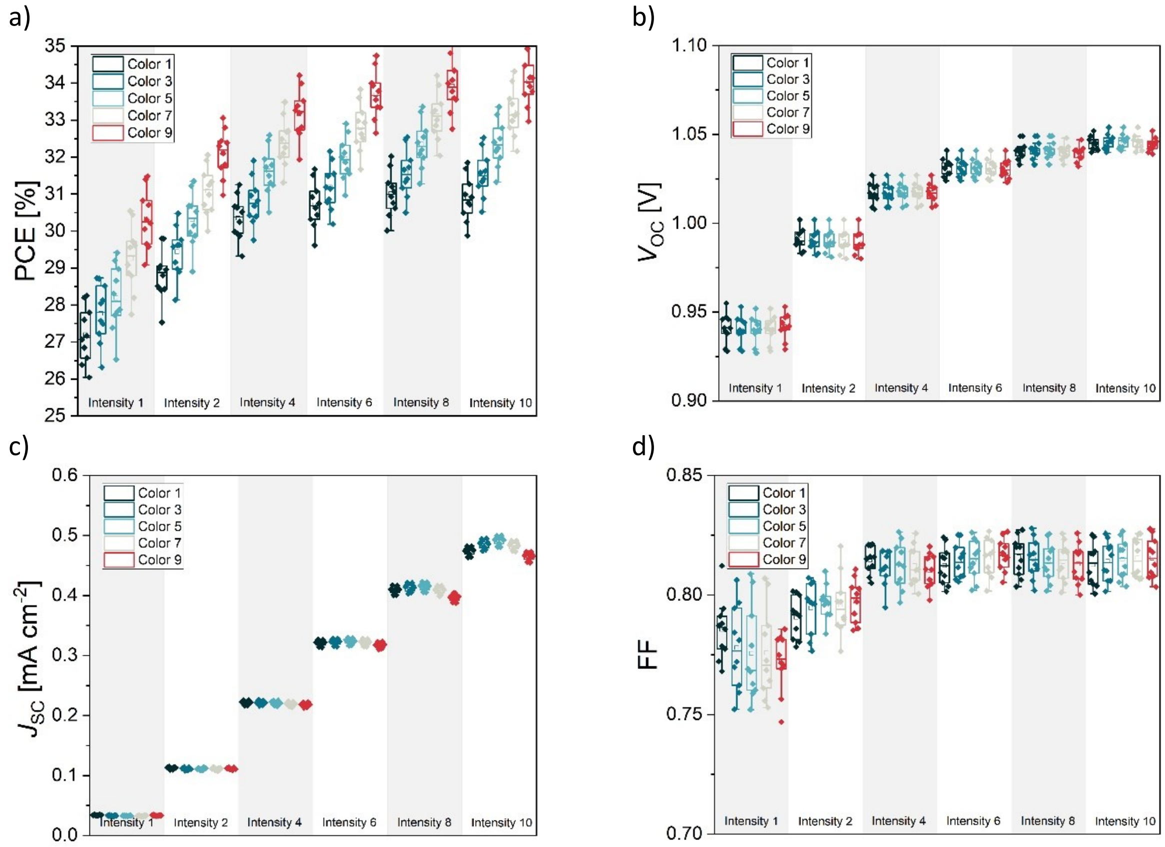

Figure S5: PV performance parameters (a) PCE, b) V_{OC}, c) J_{SC} and d) FF) of a perovskite solar cell with a bandgap of 1.68 eV under different low light intensities and spectra of the used LED lamp

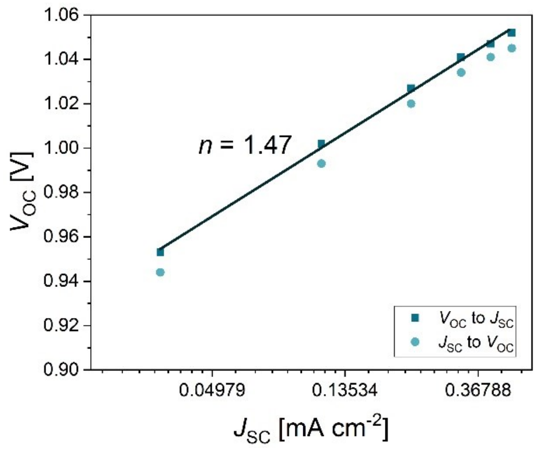

Figure S6: V_{OC} vs J_{SC} relation obtained from the measurements shown in Figure S5.

Degradation and light soaking loss during MPP tracking

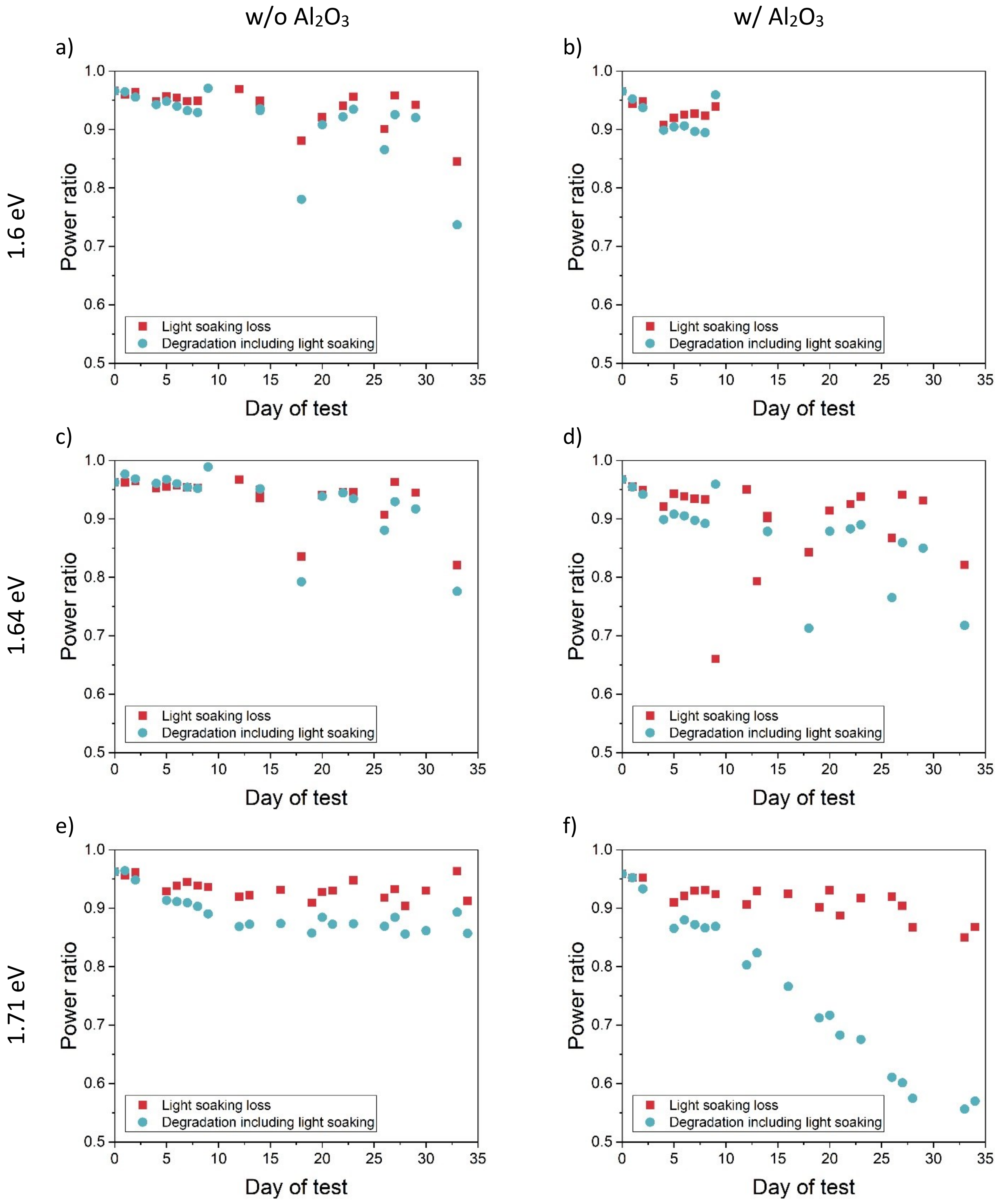

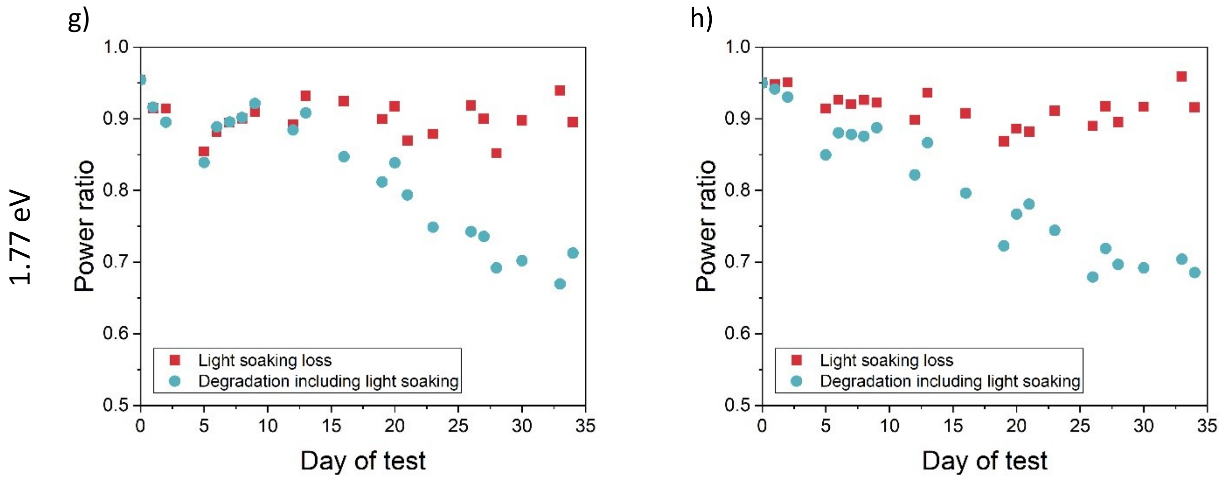

Figure S7: Daily dependent degradation and light soaking loss during the MPP cyclic testing for devices with a bandgap of: a) and b) 1.6 eV, c) and d) 1.64 eV, e) and f) 1.71 eV and g) and h) 1.77 eV. The left column, a), c), e) and g), represents devices without Al_2O_3 passivation and the right column, b), d), f) and h), devices with Al_2O_3 passivation as indicated in the figure. Daily energy is obtained by integration all power points during each day. Full degradation is obtained by dividing daily energy with the energy on day 1. Light soaking loss is obtained by dividing daily energy with expected daily energy, where we considered constant power throughout the day. Graphs start at 0.96 due to some noise in MPP tracking when measuring µA. In panel b) points after day 10 were not useful due to high noise caused by bad contact between the cell and the sample holder. Degradation typically starts at around day 10, especially for devices with Al_2O_3 passivation.
